# The Effectiveness of Direct Oral Anticoagulation in the Treatment of Left Ventricular Thrombus in Noncompaction Cardiomyopathy: A Case Report

**DOI:** 10.1002/ccr3.70583

**Published:** 2025-06-27

**Authors:** Ossama Maadarani, Leila Bigdelu, Harikrishna Rajendran, Zouhair Bitar

**Affiliations:** ^1^ Critical Care Unit, Internal Medical Department Ahmadi Hospital, Kuwait Oil Company (KOC) Al Ahmadi Kuwait; ^2^ Vascular and Endovascular Surgery Research Center, Mashhad University of Medical Sciences Mashhad Iran; ^3^ Radiology Department Ahmadi Hospital, Kuwait Oil Company Al Ahmadi Kuwait

**Keywords:** direct acting oral anticoagulant, noncompaction cardiomyopathy, thrombus

## Abstract

The use of direct oral anticoagulants (DOACs) in the treatment of left ventricular thrombus (LVT) associated with specific types of cardiomyopathy like noncompaction cardiomyopathy (NCCM) is not established. We report a case of LVT in an NCCM patient treated with DOAC with complete resolution. To the best of our knowledge, this is the second case of successful treatment of LVT related to NCCM with DOACs.

## 1 Introduction

Noncompaction cardiomyopathy (NCCM) is a rare type of cardiomyopathy where the myocardium of the left ventricle consists of two layers: a thick, sponge‐like noncompacted trabecular endocardial layer and a thin, compacted myocardial layer. This type of cardiomyopathy has a genetic origin and is characterized by prominent trabeculae and deep intertrabecular recesses that maintain communication with the ventricular cavity. Deep recesses can cause a slow, sluggish flow of blood and, combined with left ventricular systolic dysfunction, may significantly induce a hypercoagulable state with possible sequels of thrombus formation. The use of direct oral anticoagulation in treating left ventricular thrombus (LVT) associated with noncompaction cardiomyopathy has not been studied. Here, we report a case of LVT in a patient with established noncompaction cardiomyopathy that was treated with DOACs with complete resolution.

## 2 Case Report

### 2.1 Case History and Examination

A 52‐year‐old woman with no previous medical problems presented with symptoms of heart failure for a few weeks. She denied chest pain at any time. She is a nonsmoker; she denied taking any medications, recreational drugs, or alcohol and had no history of allergies. Her family history was unremarkable for heart disease and sudden cardiac death. No history of any genetic or hereditary diseases was reported in her family. Upon examination, she was mildly dyspneic; her vital signs showed a blood pressure of 110/70, a heart rate of 100 beats per minute (B/min), and oxygen saturation of 95% on room air. Her physical examination showed mild bilateral pitting edema of the lower limb and bilateral basal crepitation on both lungs filed on auscultation in addition to a systolic murmur 2/6 at the apex.

### 2.2 Investigation

**FIGURE 1:**
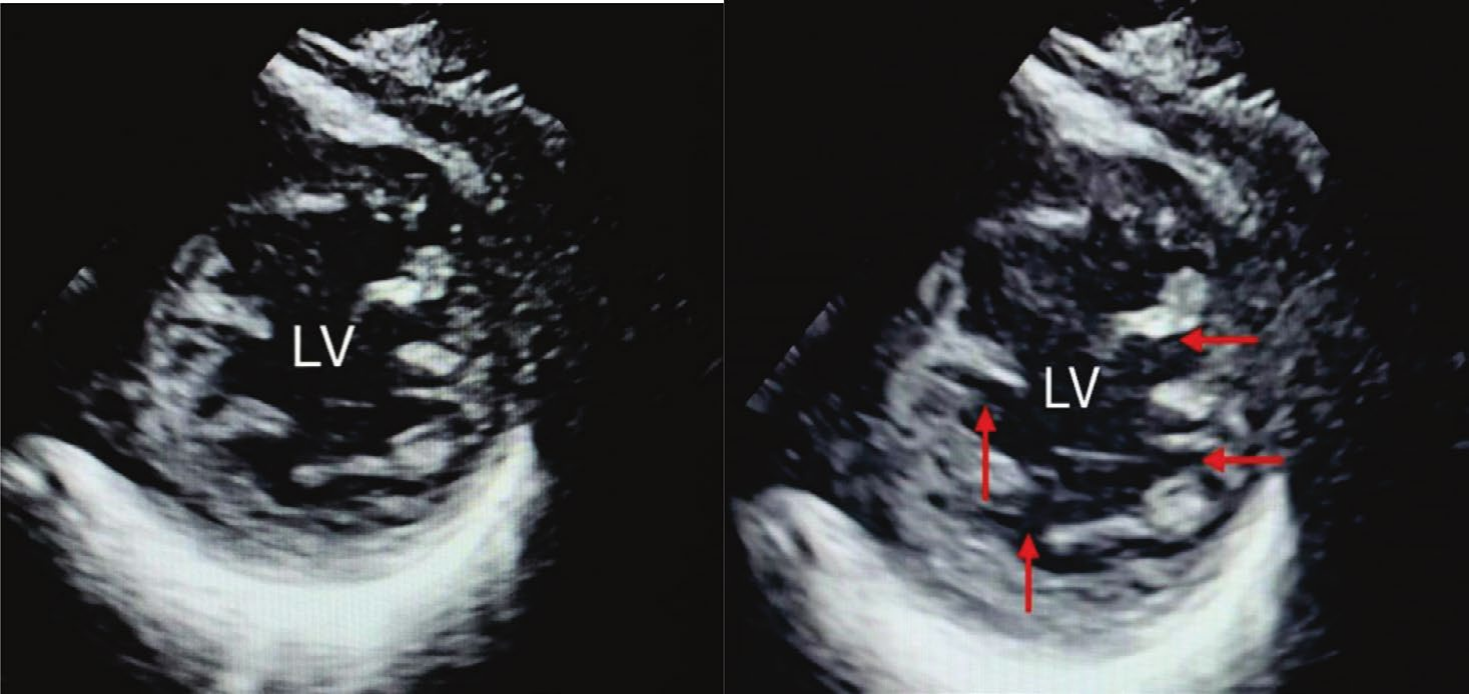
Transthoracic echocardiography: Parasternal short axis view: Prominent left ventricle (LV) trabeculations with deep recesses. The ratio of thickness of non‐compacted:compacted layer > 2:1.

**FIGURE 2:**
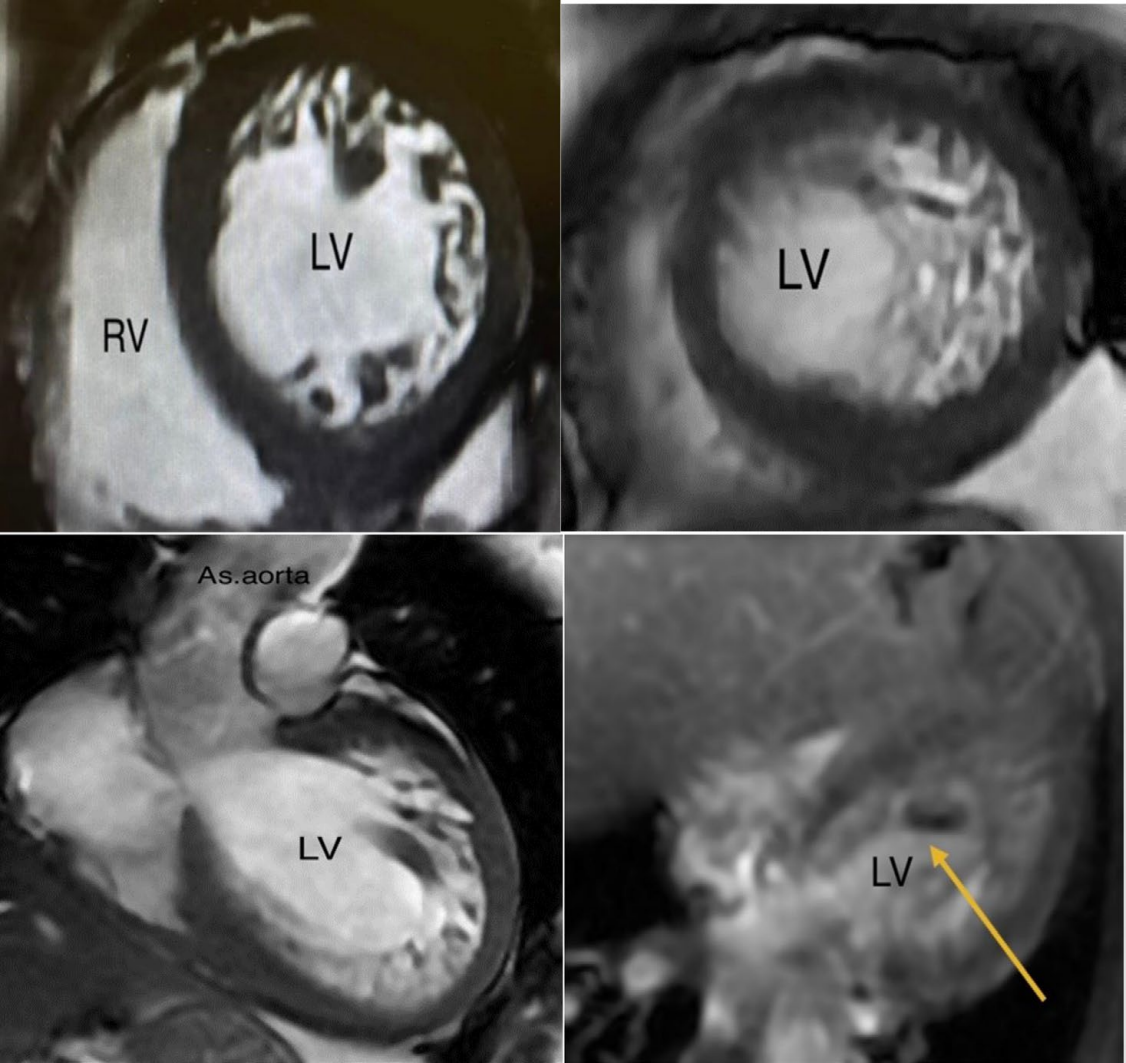
Cardiac magnetic resonance axial and four‐chamber view showing noncompaction pattern and left ventricular thrombus (yellow arrow). AS.aorta—ascending aorta, LV—left ventricle, RV—Right ventricle.

The electrocardiography (ECG) showed sinus rhythm with left bundle branch block with no evidence of ongoing ischemia, according to Smith‐modified Sgarbossa criteria. Chest X‐ray showed cardiomegaly and evidence of lung congestion. Her serial troponin measurements were non‐significantly elevated, whereas her N‐terminal pro‐B‐type natriuretic peptide was significantly high. Her laboratory investigation showed no anemia with normal renal and liver function tests. Her thyroid function and iron study were also normal. Her transthoracic echocardiography (TTE) showed a dilated left ventricle (LV) with a significantly reduced ejection fraction of 25% and global severe hypokinesia of LV with prominent trabeculations and deep recesses. The ratio of the thickness of the noncompacted to compacted (NC/C) layer was > 2:1, suggestive of a picture of noncompaction (Figure 1). No clear masses or thrombi inside LV were detected on TTE. There was mild to moderate mitral regurgitation. Cardiac magnetic resonance (CMR) showed a picture of dilated LV and hypertrabeculation with NC/C ratios of 3.7 and 2.8 at the LV apex and mid cavity, respectively. A 12 × 10 mm thrombus was detected in the posteroinferior wall, enclosing a relation with the noncompaction part of LV (Figure 2). Rest perfusion and late gadolinium enhancement scan showed no evidence of perfusion defect and no delayed myocardial enhancement.

### 2.3 Differential Diagnosis

Based on imaging modalities, the patient was diagnosed with a case of heart failure related to noncompaction cardiomyopathy with LV thrombus. She was started on classical guideline‐directed medical therapies (GDMT) for heart failure and reduced systolic function. Regarding anticoagulation therapy for LV thrombus, the decision was to start her on DOACs (Apixiban 5 mg twice daily) with a follow‐up with CMR after three months.

### 2.4 Follow‐Up

**FIGURE 3:**
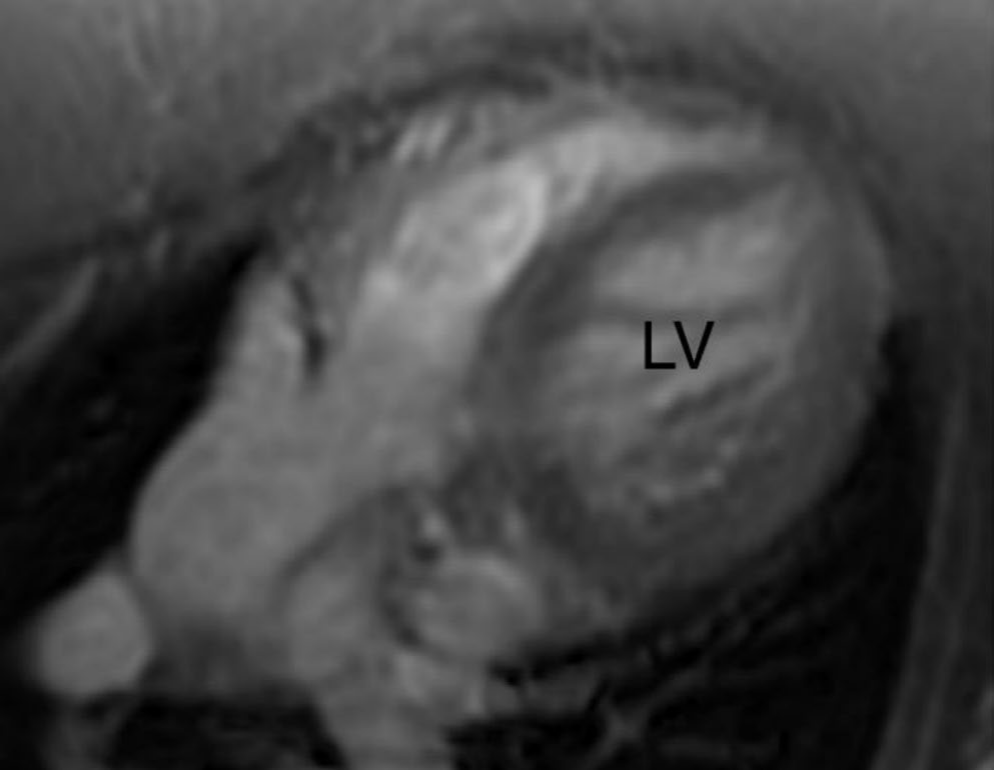
Cardiac magnetic resonance showing absence of left ventricle (LV) thrombus after treatment with apixiban.

On follow‐up, her clinical condition significantly improved with medication, and the control CMR after three months showed no evidence of thrombus in LV with improved systolic function of LV (Figure 3). The decision was to maintain the patient on a prophylactic dose of DOACs for a longer time since this condition is associated with a hypercoagulable state. To our knowledge, this is the second case of successful treatment of LVT related to NCCM with DOACs.

## 3 Discussion

Noncompaction cardiomyopathy was first characterized by Feldt et al. in 1969 [1], where they reported a biventricular spongy myocardium in a female patient. NCCM is a complex, clinically and genetically heterogeneous disorder characterized by a two‐layered myocardium: an abnormally thick and sponge‐like noncompacted trabecular layer and a thin, compacted myocardial layer that is usually associated with prominent trabeculae and deep intertrabecular recesses that keep communication with the ventricular cavity. LV noncompaction is a rare primary cardiomyopathy that may be caused by an arrest in the normal embryogenesis of the endocardium and mesocardium, leading to the formation of prominent trabeculations and deep intertrabecular recesses within the LV wall, which communicate with the cavity. The most involved segments of LV are apical and lateral segments. However, both ventricles can be affected. Among adults referred to the echocardiography laboratory, the prevalence of NCCM is estimated to be around 0.05%–0.27%; however, the exact prevalence is unknown [2], and the time of diagnosis can be at any age from childhood to late adulthood, where males appear to be more affected. In other cohorts of patients with cardiac pathology, the prevalence of LV noncompaction ranges from 0.9% by echocardiographic criteria to 9.8% with CMR criteria [3].

The genetic basis of this cardiomyopathy is associated with mutations in more than 40 genes coding for sarcomeric, cytoskeletal, Z‐line, and mitochondrial proteins [4]. Cases of NCCM have been related to genetic syndromes, including congenital heart disease, neuromuscular disorders, and facial dysmorphisms. Clinical manifestations of NCCM can range from asymptomatic status to end‐stage heart failure, serious arrhythmias, sudden cardiac arrest, or events of thromboembolism, including stroke, mesenteric, myocardial, and renal infarction, or peripheral embolism [5]. TTE and CMR are considered the most common imaging tools used for the diagnosis of NCCM, where the most commonly used criteria are the Jenni criteria [6] for TTE and the Petersen criteria [7] and the Jacquier criteria [8] for CMR.

Thromboembolic risk and hypercoagulable state in NCCM are considered critical manifestations and complications. Due to the very low prevalence of the disease, there is a lack of evidence from large, randomized trials about the clinical management of NCCM, especially regarding the need for anticoagulation. Thromboembolic risk has been suggested to result from blood stasis within the prominent LV trabeculations and intertrabecular recesses. A quite variable frequency of thromboembolism manifestation in NCCM patients ranges from 0% to 38% [9]. In a large retrospective study of 169 NCCM adult patients [10], 15% experienced thromboembolic events, mainly (92%) manifested as stroke, whereas the cardioembolic event was almost 70% and only 39% had atrial fibrillation (AF). In another study, thromboembolic events occurred in 15.3% of 144 NCCM patients, mainly manifested as stroke [11]. Long‐term follow‐up of 34 adults with isolated left ventricular noncompaction revealed thromboembolic events in eight patients (24%), as reported by Oechslin et al. Due to the rarity of NCCM and the lack of prospective trials, the risk of LVT and the mortality associated with stroke are unknown [12]. In patients with left ventricular (LV) thrombus, the current American Heart Association (AHA) and European Society of Cardiology (ESC) guidelines recommend oral anticoagulants (either DOAC or warfarin) for 3 to 6 months without any preference [13]. In a recent meta‐analysis of 4 randomized control studies, there was similar efficacy and safety of DOAC and warfarin in patients with LV thrombus [13]. Since 2010, DOACs have become widely available, but these drugs have only recently been studied for the treatment of LVT. The DOACs were not studied in the specific setting of LVT in NCCM patients. More challenging is the assessment of thromboembolic risk in NCCM patients without AF, LV dysfunction, or LVT. The AHA still recommends long‐term VKA administration as the most commonly adopted therapeutic strategy in patients with NCCM and LVT [14]. Furthermore, there is only one case report in the literature regarding the treatment of LVT in NCCM patients with DOACs. This report, presented by Sun et al., discussed the case of a 43‐year‐old patient treated with a low dose of rivaroxaban (10 mg once daily), resulting in the resolution of the thrombus at 3‐month follow‐up. To our knowledge, this is the second case of successful treatment of LVT related to NCCM with DOACs. Probably, DOACs may offer an alternative therapeutic approach in NCCM patients with LVT and/or systolic dysfunction; however, further studies with comparative groups are warranted.

## 4 Conclusion

The use of DOACs in treating LV thrombus in noncompaction cardiomyopathy could be an alternative to traditional vitamin K antagonists; however, more studies with comparative groups are necessary.

## Author Contributions

**Ossama Maadarani:** conceptualization, data curation, writing – review and editing. **Leila Bigdelu:** data curation, supervision, writing – review and editing. **Harikrishna Rajendran:** investigation, supervision. **Zouhair Bitar:** data curation, supervision, writing – original draft.

## Consent

Written informed consent for publication was obtained from the patient.

## Conflicts of Interest

The authors declare no conflicts of interest.

## Data Availability

The data that supports the findings of this case report are available from corresponding author upon request.
